# Quantitative aortic Na[^18^F]F positron emission tomography computed tomography as a tool to associate vascular calcification with major adverse cardiovascular events

**DOI:** 10.1007/s00259-024-06901-9

**Published:** 2024-09-19

**Authors:** T. G. F. Lieverse, G. D. van Praagh, D. J. Mulder, H. J. Lambers Heerspink, J. M. Wolterink, R. H. J. A. Slart

**Affiliations:** 1 Department of Clinical Pharmacy and Pharmacology, University of Groningen, University Medical Center Groningen, Groningen, The Netherlands; 2 Medical Imaging Center, Department of Nuclear Medicine and Molecular Imaging, University of Groningen, University Medical Center Groningen, Groningen, The Netherlands; 3 Department of Internal Medicine, Division of Vascular Medicine, University of Groningen, University Medical Center Groningen, Groningen, The Netherlands; 4 Department of Applied Mathematics and Technical Medical Center, University of Twente, Enschede, The Netherlands; 5 Department of Biomedical Photonic Imaging, Faculty of Science and Technology, University of Twente, Enschede, The Netherlands

**Keywords:** Na[^18^F]F, PET/CT, Agatston score, MACE, CVD

## Abstract

**Purpose:**

Sodium[^18^F]fluoride (Na[^18^F]F) used in positron emission tomography (PET) binds to active calcification and correlates consistently with higher cardiovascular risk. This study aims to investigate the feasibility of aortic Na[^18^F]F-PET in hybrid combination with low-dose computed tomography (CT) as a risk model for major adverse cardiovascular events (MACE).

**Methods:**

Patient data and Na[^18^F]F-PET/CT scans from January 2019 to February 2022 were retrospectively collected at the University Medical Center Groningen (UMCG), the Netherlands. MACE-outcome was a composite of time to first documented myocardial infarction, cerebral vascular accident (CVA), acute heart failure hospitalization, and aortic aneurysms. MACE dates were recorded from the day of the scan until follow-up in December 2023. The aorta was manually segmented in all low-dose CT scans. To minimize spill-over effects from the vertebrae, the vertebrae were automatically segmented using an open-source model, dilated with 10 mm, and subtracted from the aortic mask. The total aortic Na[^18^F]F corrected maximum standardized uptake value (cSUV_max_) and total aortic Agatston score were automatically calculated using SEQUOIA. Kaplan–Meier and Cox regression survival analysis were performed, stratifying patients into high, medium, and low cSUV_max_ and Agatston categories. Cox regression models were adjusted for age.

**Results:**

Out of 280 identified scans, 216 scans of unique patients were included. During a median follow-up of 3.9 years, 12 MACE occurred. Kaplan–Meier survival analysis demonstrated a significant difference in MACE-free survival among the high cSUV_max_ group compared to the medium and low groups (*p* = 0.03 and *p* < 0.01, respectively). Similarly, patients with high Agatston scores had a significantly lower MACE-free survival probability compared to those with medium and low scores (both *p* < 0.01).

**Conclusion:**

This study highlights the potential clinical utility of Na[^18^F]F-PET/CT as an imaging tool to predict the risk of MACE. Clinical validation of this novel proof-of-concept method is needed to confirm these results and expand the clinical context.

**Supplementary Information:**

The online version contains supplementary material available at 10.1007/s00259-024-06901-9.

## Introduction

Although primary and secondary prevention of major adverse cardiovascular events (MACE) has improved over the years, optimization and stratification of residual risk is still an important unmet medical need. Cardiovascular disease (CVD) is predominantly the clinical manifestation of atherosclerosis, a chronic inflammatory condition starting with the formation of fibrofatty lesions in the arterial wall. Calcium mineralization then promotes and solidifies plaque formation, while on the other hand microcalcification in the fibrous cap may increase local tissue stress, resulting in plaque instability [1–4].

Noninvasive imaging techniques play a central role in the identification, stratification, and follow-up of atherosclerosis in major vascular regions, such as the coronary arteries and carotid arteries. In clinical settings, non-contrast-enhanced cardiac computed tomography (CT) scans are widely used for risk profiling of (asymptomatic) individuals by calculating coronary artery calcium score, i.e., the Agatston score [5]. However, CT primarily captures vascular plaques at a relatively late stage of atherosclerotic progression, while the risk of vascular plaque rupture might occur at any time of the atherosclerotic process.[6]. Using cardiac CT, it is difficult to differentiate between vulnerable plaques and stable plaques [6]. Identifying vulnerable plaques, which are prone to cause MACE, would give an opportunity to identify and treat patients in an earlier stage [7].

**Fig. 1:**
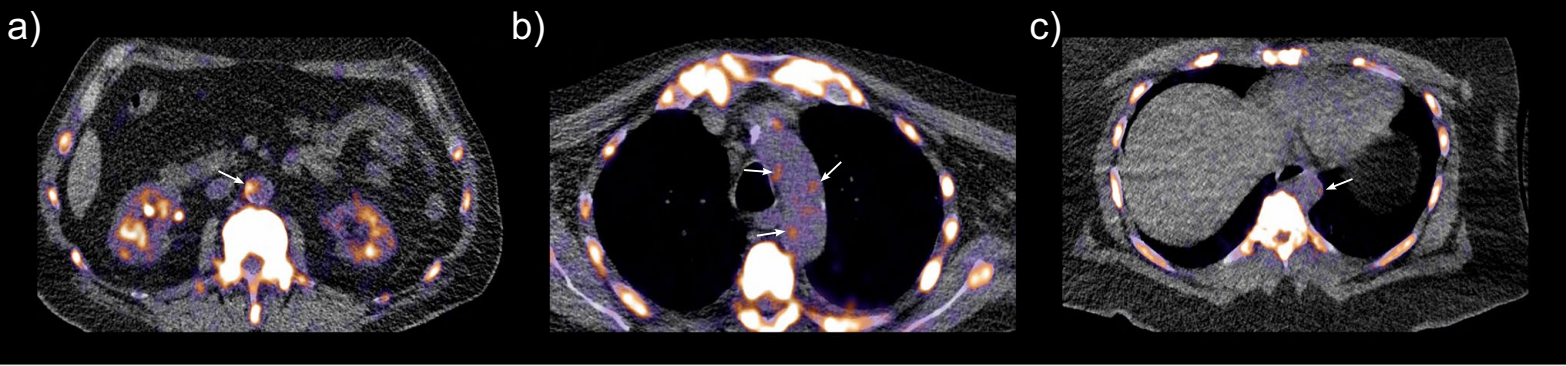
Examples of Na[^18^F]F uptake on PET and related to macrocalcifications on CT. **a** Na[^18^F]F uptake adjacent to a macrocalcification on CT; **b** Na[^18^F]F hotspots in the vascular wall of the aortic arch, but not near any macrocalcifications; **c** a patient with no calcifications, but some Na[^18^F]F hotspots in the vascular wall

Sodium [^18^F]fluoride positron emission tomography (Na[^18^F]F PET) has been demonstrated to bind to active calcification formation in the arterial vasculature [8–10]. Na[^18^F]F PET/CT is currently used for staging, restaging, and evaluation of therapy response for cancers, including bone, breast and prostate cancer [11, 12]. However, many studies demonstrated that an increased uptake of Na[^18^F]F correlates consistently with cardiovascular risk markers and is associated with a higher cardiovascular risk [13–15]. This suggests that Na[^18^F]F-PET/CT may provide an earlier assessment of the atherosclerotic burden in the body than cardiac CT. Figure 1 illustrates various examples of Na[^18^F]F uptake in relation to macrocalcifications on CT. Furthermore, previous studies are primarily concentrated on the coronary arteries and the aortic valve, while small lesions in the coronary arteries are difficult to visualize on low-dose CT used in PET/CT [3, 15, 16]. Evaluating vascular calcification in the aorta could therefore offer additional predictive information [17]. Additionally, the focus in the majority of studies for assessing cardiovascular risk is predominantly centered around the evaluation of coronary events [18, 19]. Using MACE to study cardiovascular risk could therefore offer a more comprehensive risk analysis [20]. Moreover, studies that examined the feasibility of the Na[^18^F]F-PET/CT scan frequently included patients who had preexisting CVD, rendering it difficult to perform a comprehensive risk analysis for CVD in this context [14, 19]. Therefore, the aim of this study is to investigate the feasibility of using aortic hybrid Na[^18^F]F-PET/CT as a risk model for MACE.

## Material and methods

### Patient data

All patients that received a Na[^18^F]F-PET/CT scan between January 2019 and February 2022 at the UMCG were included in this study. This consisted of scans acquired for screening of staging, restaging, and evaluation of therapy response for bone, breast or prostate cancer, and scans acquired from diabetes mellitus type 2 patients from the DETERMINE cohort. Inclusion criteria of that cohort have been published before [21]. The Na[^18^F]F-PET/CT scans were retrieved from the PACS together with patient data from the electronic patient file system. Follow-up scans of the same patients were excluded. The study was conducted in compliance with the principles of the Declaration of Helsinki. The Medical Ethical Institutional Review Board of the University Medical Center Groningen (UMCG, Research Register number: 201800548, METc number 2018.456) approved the protocol. All participants gave written informed consent. For all other scans, due to the retrospective design, the Medical Research Involving Human Subjects Acts (WMO) obligation was waived by the local ethics committee (registration no. METC 2021/173). The objection registry was checked for all patients, but no informed consent was obtained.

Our primary outcome measure was the incidence of MACE starting from the date of the Na[^18^F]F-PET/CT scan until follow-up in December 2023. This was investigated by a single observer. MACE was defined as the composite of myocardial infarction, cerebral vascular accident (CVA), hospitalization due to acute heart failure, and the first detection of aortic aneurysms, based on current guidelines and literature [4, 20, 22–27]. Myocardial infarction, acute heart failure and CVAs were defined based on the guidelines of the American College of Cardiology and the American Heart Association [28–32]. Acute heart failure was included as a MACE endpoint only if it was attributable to an ischemic cause or if the patients had a known history of vascular disease or angina pectoris. CVAs included both ischemic strokes and hemorrhagic strokes. Aortic aneurysms were defined based on the guidelines on the Society of Vascular Surgery, the American Heart Association, and the American College of Cardiology [33, 34]. As our dataset predominantly consisted of oncology patients, death was not included as a MACE in the main analysis, making it difficult to differentiate between oncological and cardiovascular death. Baseline data included age, sex, medical history pertaining to the occurrence of MACE after the Na[^18^F]F-PET/CT scan, estimated glomerular filtration rate (eGFR), and body mass index (BMI).

### Scan acquisition and reconstruction

Scans were acquired on the following PET/CT scanners: Biograph mCT40, Biograph mCT64, or Biograph Vision (Siemens Healthineers, Knoxville, TN, USA). Na[^18^F]F-PET/CT scans were performed following the European Association of Nuclear Medicine (EANM) procedure guidelines [35, 36]. All PET/CT scanners employed have obtained EANM Research Ltd. (EARL) accreditation. After at least six hours of fasting, two (for Vision) or three (for mCT) MBq/kg of tracer was intravenously administered to the patient. Sixty minutes after administration, a low-dose CT and subsequent PET scan from skull to knee, 1–2 min per bed position, were performed. Scans were then reconstructed according to the EANM research life (EARL) guidelines [37].

### Aortic analysis

The aorta was manually delineated on the low-dose CT using Affinity Viewer (version 2.0.3, Hermes Medical Solution, Stockholm, Sweden) from the slice just above the aortic valve up to the iliac bifurcation. Background measurements were performed by drawing three spherical volumes of interest of 1 mL each in the center of the superior vena cava. When imaging artefacts were present in the superior vena cava due to, for example, pacemaker leads, the measurements were performed in the inferior vena cava. As Na[^18^F]F uptake in bones is substantially higher than in vessels, spill-over from the vertebrae into the adjacent aorta needed to be excluded. Therefore, the vertebrae were automatically segmented using a publicly available convolutional neural network [38]. After examination, these vertebral segmentations were closed and dilated by 10 mm and then subtracted from the aortic mask. This method was previously described [39]. By overlaying the resulting aortic mask onto the PET, the blood pool corrected maximum standardized uptake value (cSUV_max_) was calculated to represent the total aortic calcium burden. The calculation of the cSUV_max_ was proposed by Blomberg et al. in the CAMONA study as a better reproducible metric compared to the target-to-background ratio [40]. Briefly, the SUV_max_ of every slice of the aortic mask was summed and divided by the total number of slices (average SUV_max_). The SUV_mean_ of the three background measurements (blood pool) was then subtracted from this average SUV_max_ to obtain the cSUV_max_.

Additionally, the Agatston score was calculated. In the aortic mask overlayed onto the low-dose CT a standard 130 Hounsfield unit threshold was applied. Calcified lesions were defined as connected pixels > 1 mm^2^. The Agatston score was calculated in each lesion and summed to obtain the total aortic Agatston score. The cSUV_max_ and Agatston score were calculated using SEQUOIA [41].

### Statistical analysis

Normally distributed data was presented as mean ± standard deviation (SD) and non-parametric data as median [interquartile range (IQR)]. Kaplan–Meier and Cox regression survival analyses were performed to assess the association between cSUV_max_ and MACE rates, as well as between the Agatston score and MACE rates. The initiation of the study period is marked by the date of the Na[^18^F]F-PET/CT scan, with the conclusion being determined by the occurrence of a MACE or the designated follow-up date. For the Kaplan–Meier survival analyses, patients were stratified into high, medium, and low cSUV_max_ groups and high, medium, and low Agatston groups using the first and second tertiles of those respective values. A log-rank test was applied to compare survival between the three groups. Then Cox regression analyses were performed to assess the association between the two metrics and survival rate. The analyses were adjusted for age only due to limited number of events. The likelihood ratio test was performed as a general test of the overall significance of the Cox regression model. The Wald test was performed for each individual predictor variable in the Cox regression model. Furthermore, a Cohen’s kappa was calculated to find interrater agreement between the classification of total aortic cSUV_max_ and the total aortic Agatston score into the three risk groups. A p-value < 0.05 was considered statistically significant. All data management and statistical analysis were performed using GraphPad Prism version 10 (GraphPad Software, Boston, MA, USA).

## Results

**Fig. 2:**
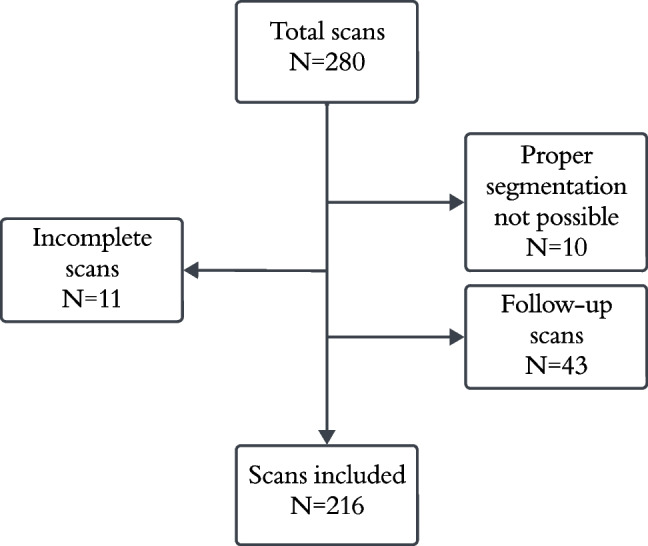
Flow diagram of scan inclusion

We identified 280 Na[^18^F]F-PET/CT scans. After exclusion of incomplete and follow-up scans from the dataset, 216 patients and corresponding Na[^18^F]F-PET/CT scans were included (Fig. 2). 186 Na[^18^F]F-PET/CT scans were retrieved from patients who were screened for staging, restaging, and evaluation of therapy response for bone, breast or prostate cancer in the UMCG. Thirty scans were retrieved from the prospective DETERMINE cohort [21]. The baseline patient characteristics together with the PET/CT acquisition parameters and cSUV_max_ and Agatston tertiles are shown in Table 1. The baseline characteristics of both patient groups are provided separately in Supplementary Table 1. An overview about the demographics of the three patient groups stratified according to cSUVmax and Agatston tertiles are given in Supplementary Tables 2 and 3. The total patient population consisting of 216 individuals included 119 males (55%) with a mean age and range of 61 [7 – 92] years. Median follow-up until December 2023 was 3.9 [1.8 – 5.9] years. During follow-up, in total twelve unique occurrences of MACE across multiple patients were counted. Two patients experienced a myocardial infarction, three patients experienced a CVA, two patients were admitted to the hospital with acute heart failure, four patients developed an aortic aneurysm, and one patient suffered from both a CVA and acute heart failure that required hospitalization (Table 2).

**Table 1:** Patient characteristics and PET/CT acquisition parameters

Characteristics	*N* (%) or mean ± SD or median (IQR)
No. of patients	216
Age on date of scan (years) [range]	61 ± 17 [7– 92]
Sex-type (males)	119 (55%)
BMI (kg/m^2^) [range]	24.5 ± 9.5 [15.1 – 43.2]^†^
Estimated GFR (mL/min/1.73m^2^)	79 ± 27 [10 – 142]^ƒ^
Scanners	
Biograph mCT40	53 (25%)
Biograph mCT64	63 (29%)
Biograph Vision	100 (46%)
Tube voltage (kV)	80 (N = 3), 100 (N = 15), 120 (N = 159), 140 (N = 39)
Tube current time product (mAs)	11.5 (8.5 – 18.6)
Slice thickness (mm)	3
Matrix size (pixel x pixel)	512 × 512
cSUV_max_	
Median [first – second tertile]*	0.64 [0.50 – 0.77]
Range	0.05 – 1.87
Agatston score	
Median [first – second tertile]*	2893 [1403 – 7153]
Range	0—79078

^†^Of 21 patients, length and/or weight was unknown

^ƒ^Of 18 patients, estimated GFR was unknown

^*^Tertiles were used to define low, medium, and high groups

*SD* standard deviation; *IQR* interquartile range; *BMI* body mass index; *GFR* glomerular filtration rate; *cSUV*_*max*_ blood pool corrected maximum standardized uptake value

**Table 2:** MACE occurrence until December 2023

MACE	N of patients experiencing MACE	% of total patients
Myocardial infarction	2	0.9%
CVA	4	1.9%
Acute heart failure	2	0.9%
Aortic aneurysm	4	1.9%

**Fig. 3:**
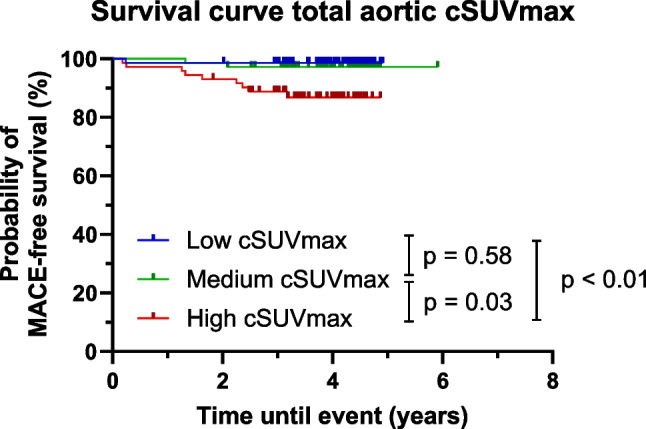
Survival curve based on the cSUV_max_ in patients who underwent Na[^18^F]F-PET/CT scans

The high cSUV_max_ group showed a significant difference in MACE-free survival compared with the medium and low groups (*p* = 0.03 and *p* < 0.01, respectively) (Fig. 3). No significant difference was found between the medium and low groups (*p* = 0.58). Nine of the MACE occurred in patients from the highest cSUV_max_ group. Two of the MACE occurred in patients from the medium group and one in a patient from the low group. Higher cSUV_max_ was associated with a significantly higher risk of MACE events after adjustment for age (*p* < 0.001).

**Fig. 4:**
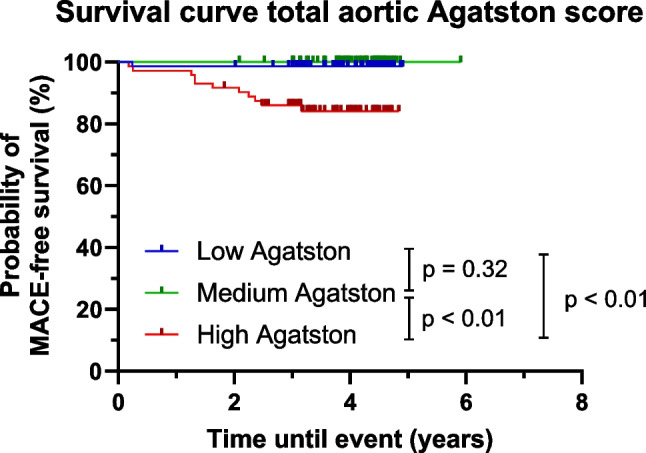
Survival curve based on the total aortic Agatston score in patients who underwent Na[^18^F]F-PET/CT scans

Similar survival curves were found for the total aortic Agatston score (Fig. 4). The high Agatston group had a significantly reduced MACE-free survival probability compared with the medium and low group (both *p* < 0.01). No significant difference was found between the medium and low groups (*p* = 0.32). Eleven of the MACE events occurred in patients from the highest Agatston score group. Zero MACE events occurred in patients from the medium group, and one event occurred in a patient from the low group. Higher Agatston scores were associated with a significantly higher risk of MACE events after adjustment for age (*p* < 0.001).

**Fig. 5:**
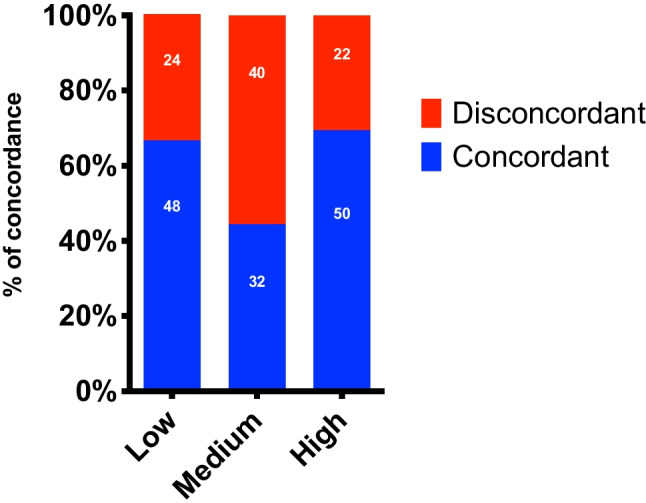
Bar chart that visualizes the concordance and discordance between the cSUVmax and Agatston score across the different risk tertiles. The numbers within the bars represent the sample sizes

A moderate agreement was observed between risk classification of the total aortic cSUV_max_ and total aortic Agatston score (κ = 0.468 [-0.054–0.990]). The concordance of patients between cSUV_max_ and Agatston groups was 60.2% in total. Per group this was 66,7% for the low group, 44,4% for the medium group, and 69,4% for the high group (Fig. 5).

## Discussion

This study aimed to evaluate the feasibility of utilizing aortic Na[^18^F]F-PET/CT as a risk model for the emergence of MACE. The main results revealed a significant association between the predictive value of the total aortic cSUV_max_ in hybrid Na[^18^F]F-PET/low-dose CT scans, and the subsequent emergence of MACE during an approximately 4-year follow-up period. Survival analyses were comparable to the total aortic Agatston score and revealed a notable one-year survival rate, reflecting the early detection capability of Na[^18^F]F-PET/CT in identifying cardiovascular disease at its early stages. In this mostly oncological and elderly patient population, the predictive value of cSUV_max_ for MACE was comparable to the aortic Agatston score. These findings highlight the potential clinical utility of Na[^18^F]F-PET/CT as an imaging tool to predict the risk of MACE.

As stated before, the Agatston score is widely considered the gold standard for CT calcium scoring in cardiac CT scans in the clinical setting. Therefore, to examine the feasibility of the Na[^18^F]F-PET/CT scan as a risk model for MACE, both the cSUV_max_ and the Agatston score were incorporated for comparative purposes. Low-dose CT from PET/CT is not currently utilized in clinical risk modeling for MACE. However, by comparing patients in three groups rather than individually, our study provides a preliminary step towards integrating Na[^18^F]F-PET/CT into clinically established risk models for MACE. Besides, comparable with previous studies, which examined the predictive value of Na[^18^F]F uptake in coronary artery disease, we found a similar predictive value of the cSUV_max_ as well as the Agatston score [14, 18, 19]. However, the clinically added predictive value of Na[^18^F]F is due to its potential to provide earlier assessment of the atherosclerotic burden than currently used cardiac CT. This study demonstrates this predictive value of Na[^18^F]F for MACE in a relatively small cohort and short follow-up. Moreover, we believe this data contributes to the understanding of its role in risk assessment, especially in populations where traditional methods might lack the necessary sensitivity to detect subtle yet significant variations in cardiovascular risk. Large, longitudinal, prospective cohorts are needed to investigate its exact predictive value.

To the best of our knowledge, this is the first study to have evaluated the feasibility of utilizing aortic Na[^18^F]F-PET/CT as a risk model for MACE. Previous studies have investigated Na[^18^F]F uptake in the aorta. However, their focus did not include evaluating MACE or overall cardiovascular risk. For instance, Forsythe et al. and Syed et al. examined Na[^18^F]F uptake in acute aortic syndrome and abdominal aortic aneurysms, respectively [42, 43]. In contrast, Fiz et al. retrospectively explored the associations between baseline Na[^18^F]F uptake and the progression of atherosclerotic plaques within the abdominal aorta [44]. Fletcher et al. specifically explored the use of thoracic Na[^18^F]F PET to identify patients at the highest risk of ischemic stroke by using patients with stable CVD [45]. While Fletcher et al.’s findings are significant within the context of ischemic stroke, our study broadens the scope by encompassing various adverse cardiovascular events and patients without previously diagnosed CVD, providing a more comprehensive assessment of cardiovascular risk. Notably, all these studies demonstrated a positive correlation between Na[^18^F]F uptake and the occurrence of disease or the evolution of atherosclerotic plaques [42–45]. By incorporating analysis of aortic Na[^18^F]F uptake, we aimed to provide a more comprehensive evaluation of the risk of MACE, complementing the assessment of coronary arteries.

Other studies are primarily focused on assessing Na[^18^F]F uptake in the coronary arteries and the aortic valve [3, 14, 15, 18, 19, 46, 47]. However, molecular imaging of coronary artery atherosclerosis poses challenges due to constant cardiac and respiratory movements, making it difficult to accurately evaluate focal uptake of targeted tracers at the intended sites [16]. Recent research suggests that exploring vascular calcification in the aorta could provide additional predictive information for cardiovascular risk assessment [17]. Additionally, although carotid and femoral arteries are also critical sites for atherosclerotic plaque buildup, we did not include them due to technical limitations. Specifically, low-dose CT, which was used in our hybrid imaging approach, does not provide sufficient image quality to accurately distinguish and segment these smaller arteries. Future studies may benefit from using higher resolution imaging modalities to include carotid and femoral arteries, for a more comprehensive evaluation of systemic atherosclerosis.

Furthermore, the methodologies employed in previous studies for assessing cardiovascular risk are predominantly centered around the evaluation of coronary events. Although these studies demonstrated a similar outcome, the implementation of a composite endpoint such as MACE has become increasingly prevalent, given the growing emphasis on a comprehensive risk assessment in CVD research [20]. In line with this trend, we incorporated the MACE composite endpoint in our study to provide a more comprehensive evaluation of overall cardiovascular risk.

A well-known limitation of cardiovascular quantification in PET/CT is the time-consuming and labor-intensive manual delineation of the entire aorta, hence making it clinically infeasible. The development of automated segmentation and quantification methods provides a clear and efficient approach to evaluating a patient's risk of experiencing a MACE [48]. Future studies should utilize these automated analysis tools to assess their clinical feasibility. This could potentially serve as a valuable tool for both initial diagnosis and ongoing vascular disease monitoring.

This study has its limitations. First, this was a retrospective study. Second, it should be noted that the patient group employed in this study largely consisted of individuals diagnosed with bone, breast or prostate cancer, rather than the ideal group of individuals solely at risk of CVD. With this current population, patients may have succumbed to cancer-related mortality rather than experiencing a MACE. In regard to the study population, detailed information regarding the type of cancer, stage of their evolutionary metabolic disease, and their treatment regimen was not systematically collected in this study. The diverse types of cancer and their respective treatments could affect the aortic uptake of Na[^18^F]F, potentially impacting our results. For example, it is noteworthy to consider the potential influence of current standard treatments such as bisphosphonates or denosumab on the process of (micro)calcification, particularly in the context of patients with cancer-related bone pathology. These medications, commonly prescribed to mitigate bone loss and skeletal-related events in individuals with bone metastases, may inadvertently affect the dynamics of (micro)calcification, potentially confounding the interpretation of cardiovascular imaging findings [49, 50]. This heterogeneity in patient characteristics underscores the importance of considering these factors in future studies. Future research should stratify patients based on cancer type, stage, and treatment to minimize bias and improve the accuracy of the findings. Furthermore, future investigations should aim to elucidate the interplay between these treatments and cardiovascular risk markers, including the implications for (micro)calcification processes observed in imaging modalities. Third, with only twelve patients experiencing a composite cardiovascular end-point during follow-up, the robustness of the conclusions is limited. Fourth, hospitalization may have occurred at other medical institutions. Regrettably, it cannot be excluded that certain hospitalizations have been missed. However, medical events are consistently documented in the electronic patient file system which largely covers this limitation. However, the results still showed a positive and significant association of cSUV_max_ with MACE and the results were comparable with those of prior studies. Fifth, the Agatston score was calculated in the aorta in low-dose CT and not in diagnostic CT. Besides, two different scanners were used, a conventional and digital PET system. Noise most probably influenced these scores. However, we still observed differences in event rates between the three different groups. To ultimately confirm the influence of different scanners, the positive predictive value of the Na[^18^F]F-PET/CT scan, and whether Na[^18^F]F is an earlier predictor than conventional CT calcium scoring, large prospective studies are needed with patients earlier in the atherosclerotic process comparing Na[^18^F]F PET with diagnostic CT. Given the limited number of MACE events, the Cox regression analysis in this study only included age as a factor. Last, MACE was defined as the composite of myocardial infarction, CVA, the first detection of aortic aneurysms, and hospitalization due to acute heart failure. This end-point is non-specific because it includes cardiac and non-cardiac complications. However, there has not yet been a consensus about how to define MACE [20]. While myocardial infarction and CVAs are widely accepted as strong MACE criteria, consensus has not yet been reached on whether aortic aneurysms and hospitalization due to acute heart failure should be included in the MACE criteria. Establishing a standardized MACE definition is essential to reduce heterogeneity and ensure the reproducibility of future studies. Furthermore, while our composite endpoint was intended to improve the robustness of our analysis, we acknowledge that it encompasses both cardiac and non-cardiac complications, which may dilute the specificity of our findings. Future studies with larger populations are necessary to validate these specific correlations and enhance the clinical utility of Na[^18^F]F-PET/CT in predicting regional cardiovascular events.

In conclusion, the study findings highlight the potential clinical utility of Na[^18^F]F-PET/CT as an imaging tool to predict the risk of MACE. This study demonstrated a significant association between Na[^18^F]F uptake and an increased risk of MACE, comparable to the association between aortic Agatston scores and MACE. While our study provides evidence to support the feasibility of using Na[^18^F]F-PET/CT as a risk predictor for cardiovascular events, further prospective research is needed to confirm and broaden these results. Future studies should compose individuals solely at an earlier phase of CVD risk to ultimately assess the feasibility of using Na[^18^F]F-PET/CT as a risk model for MACE.

## Supplementary Information

Below is the link to the electronic supplementary material.

Supplementary file1 (DOCX 15 KB)

Supplementary file2 (DOCX 15.3 KB)

Supplementary file3 (DOCX 15.3 KB)

## Data Availability

The data underlying this article cannot be shared publicly due to the privacy of the individuals that participated in this study. The data will be shared on reasonable request to the corresponding author.
