# Supplementary material for: Quantitative aortic Na[18F]F positron emission tomography computed tomography as a tool to associate vascular calcification with major adverse cardiovascular events
Source: Eur J Nucl Med Mol Imaging. 2024 Sep 19;52(2):501–9. doi: 10.1007/s00259-024-06901-9 (PMC11732873; doi:10.1007/s00259-024-06901-9)
Supplement: Supplementary file 1 — Supplementary file1 (DOCX 15 KB) [file 259_2024_6901_MOESM1_ESM.docx]

**Supplementary Tables**

**Supplementary Table 1** Patient characteristics of patients who were screened with a Na[^18^F]F-PET/CT scan for staging, restaging, and evaluation of therapy response for bone, breast or prostate cancer

| **Characteristics** | **N (%) or mean ± SD** | | |
| --- | --- | --- | --- |
|  | **Screened for cancer** | **DETERMINE cohort** | |
| No. of patients | 186 | 30 | |
| Age on date of scan (years) [range] | 61 ± 18 [7 – 92] | 65 ± 10 [31 – 77] | |
| Sex-type (males) | 101 (54%) | 18 (60%) | |
| BMI (kg/m^2^) [range] | 26.1 ± 4.9 [15.1 – 43.2]^†^ | 32.6 ± 4.8 [23.5 – 41.2]^†^ | |
| Estimated GFR (mL/min/1.73m2) | 82 ± 16 [14 – 142]^ƒ^ | 58 ± 33 [10 – 124]^ƒ^ | |
| ^†^Of 21 patients, length and/or weight was unknown.  ^ƒ^Of 18 patients, estimated GFR was unknown.  SD = standard deviation; interquartile range; BMI = body mass index; GFR = glomerular filtration rate | | |  |
