# Supplementary material for: Quantitative aortic Na[18F]F positron emission tomography computed tomography as a tool to associate vascular calcification with major adverse cardiovascular events
Source: Eur J Nucl Med Mol Imaging. 2024 Sep 19;52(2):501–9. doi: 10.1007/s00259-024-06901-9 (PMC11732873; doi:10.1007/s00259-024-06901-9)
Supplement: Supplementary file 2 — Supplementary file2 (DOCX 15.3 KB) [file 259_2024_6901_MOESM2_ESM.docx]

**Supplementary Tables**

**Supplementary Table 2** Patient characteristics of patients who underwent Na[^18^F]F-PET/CT scans according to cSUV_max_ tertiles

| **Characteristics** | **N (%) or mean ± SD** | | |
| --- | --- | --- | --- |
|  | **Low** | **Medium** | **High** |
| No. of patients | 72 | 72 | 72 |
| Age on date of scan (years) [range] | 51 ± 19 [7 – 87] | 63 ± 12 [27 – 84] | 70 ± 13 [27 – 92] |
| Sex-type (males) | 29 (40%) | 40 (56%) | 50 (69%) |
| BMI (kg/m^2^) [range] | 25.1 ± 5.1 [15.1 – 37.0]^†^ | 28.0 ± 4.8 [17.9 – 39.9]^††^ | 28.1 ± 5.7 [18.0 – 43.2]^†††^ |
| Estimated GFR (mL/min/1.73m2) | 88 ± 24 [14 – 142]^ƒ^ | 79 ± 26 [10 – 136]^ƒƒ^ | 69 ± 26 [11 – 126]^ƒƒƒ^ |
| SD = standard deviation; interquartile range; BMI = body mass index; GFR = glomerular filtration rate | ^†^Of 9 patients, length and/or weight was unknown.  ^ƒ^Of 10 patients, estimated GFR was unknown. | ^††^Of 7 patients, length and/or weight was unknown.  ^ƒƒ^Of 6 patients, estimated GFR was unknown. | ^†††^Of 5 patients, length and/or weight was unknown.  ^ƒƒƒ^Of 2 patients, estimated GFR was unknown. |
