# Supplementary material for: Quantitative aortic Na[18F]F positron emission tomography computed tomography as a tool to associate vascular calcification with major adverse cardiovascular events
Source: Eur J Nucl Med Mol Imaging. 2024 Sep 19;52(2):501–9. doi: 10.1007/s00259-024-06901-9 (PMC11732873; doi:10.1007/s00259-024-06901-9)
Supplement: Supplementary file 3 — Supplementary file3 (DOCX 15.3 KB) [file 259_2024_6901_MOESM3_ESM.docx]

**Supplementary Tables**

**Supplementary Table 3** Patient characteristics of patients who underwent Na[^18^F]F-PET/CT scans according to Agatston tertiles

| **Characteristics** | **N (%) or mean ± SD** | | |
| --- | --- | --- | --- |
|  | **Low** | **Medium** | **High** |
| No. of patients | 72 | 72 | 72 |
| Age on date of scan (years) [range] | 48 ± 16 [7 – 79] | 62 ± 13 [17 – 83] | 74 ± 8 [57 – 92] |
| Sex-type (males) | 22 (31%) | 41 (57%) | 56 (78%) |
| BMI (kg/m^2^) [range] | 26.2 ± 5.8 [15.1 – 41.2]^†^ | 27.3 ± 5.0 [15.1 – 43.2]^††^ | 27.7 ± 5.3 [18.0 – 40.4]^†††^ |
| eGFR (mL/min/1.73m2) | 92 ± 22 [19 – 142]^ƒ^ | 79 ± 26 [10 – 132]^ƒƒ^ | 66 ± 25 [11 – 110]^ƒƒƒ^ |
| SD = standard deviation; interquartile range; BMI = body mass index; eGFR = estimated glomerular filtration rate | ^†^Of 10 patients, length and/or weight was unknown.  ^ƒ^Of 12 patients, eGFR was unknown. | ^††^Of 7 patients, length and/or weight was unknown.  ^ƒƒ^Of 5 patients, eGFR was unknown. | ^†††^Of 4 patients, length and/or weight was unknown.  ^ƒƒƒ^Of 1 patient, eGFR was unknown. |
